# Correction of thermal airflow distortion in warpage measurements of microelectronic packaging structures via deep learning-based digital image correlation

**DOI:** 10.1038/s41378-024-00764-8

**Published:** 2024-08-26

**Authors:** Yuhan Gao, Yuxin Chen, Ziniu Yu, Chuanguo Xiong, Xin Lei, Weishan Lv, Sheng Liu, Fulong Zhu

**Affiliations:** 1 Institute of Microsystems, School of Mechanical Science and Engineering, Huazhong University of Science and Technology, Wuhan, 430074 China; 2 School of Power and Mechanical Engineering, Wuhan University, Wuhan, 430072 China

**Keywords:** Electrical and electronic engineering, Micro-optics, Optical sensors

## Abstract

The projected speckle-based three-dimensional digital image correlation method (3D-DIC) is being increasingly used in the reliability measurement of microelectronic packaging structures because of its noninvasive nature, high precision, and low cost. However, during the measurement of the thermal reliability of packaging structures, the thermal airflow generated by heating introduces distortions in the images captured by the DIC measurement system, impacting the accuracy and reliability of noncontact measurements. To address this challenge, a thermal airflow distortion correction model based on the transformer attention mechanism is proposed specifically for the measurement of thermal warpage in microelectronic packaging structures. This model avoids the oversmoothing issue associated with convolutional neural networks and the lack of physical constraints in generative adversarial networks, ensuring the precision of grayscale gradient changes in speckle patterns and minimizing adverse effects on DIC calculation accuracy. By inputting the distorted images captured by the DIC measurement system into the network, corrected images are obtained for 3D-DIC calculations, thus allowing the thermal warpage measurement results of the sample to be acquired. Through experiments measuring topography with customized step block specimens, the effectiveness of the proposed method in improving warpage measurement accuracy is confirmed; this is particularly true when captured images are affected by thermal airflow at 140 °C and 160 °C, temperatures commonly encountered in thermal reliability testing of packaging structures. The method successfully reduces the standard deviation from 9.829 to 5.943 µm and from 12.318 to 6.418 µm, respectively. The results demonstrate the substantial practical value of this method for measuring thermal warpage in microelectronic packaging structures.

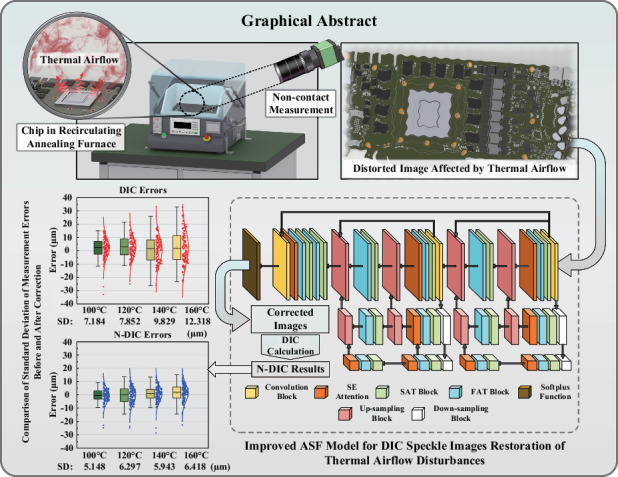

## Introduction

With the increasing integration levels and diminishing dimensions of microelectronic packaging, the demand for precise and reliable measurement methodologies to ensure the structural integrity of microelectronic packaging has significantly increased^1–4^. The heating process of chip packaging introduces a critical challenge; varying thermal expansion rates among the packaging layers can result in thermal warpage or, in severe cases, cracking, thereby severely compromising the reliability of the electronic packaging^5–8^. Against this backdrop, the projected speckle-based three-dimensional digital image correlation (3D-DIC) technique has gained prominence. This noncontact, in situ measurement method is increasingly favored for its simplicity, reliability, high precision, and noninvasive nature^9,10^, indicating that it is an indispensable tool in the domain of packaging structure warpage measurement^11–13^. Nonetheless, the precision of 3D-DIC measurements is critically hampered by the thermal airflow generated during the heating process in the reliability testing of microelectronic packaging structures. These airflows induce distortions and noise in the captured images, substantially impairing the capacity of the 3D-DIC method to measure thermal warpage with the requisite accuracy.

To evaluate the thermal reliability of microelectronic packaging structures, heating furnaces are commonly employed to heat packaging samples and observe their thermal warpage behavior^14–17^. In this process, the heating apparatus inevitably heats the air between the DIC measurement system and the sample; this leads to variations in the thermal gradients across the optical path, both spatially and temporally^18,19^. Such variations alter the refractive index of the air, an effect known as the thermal airflow effect, which results in the DIC system capturing distorted images. During rapid heating or cooling processes in a furnace, the temperature gradients are greater, leading to more significant distortions in the images. When these distorted images are analyzed with DIC algorithms, the presence of thermal airflow typically introduces substantial errors and variability in the measurements^20^. Particularly in the case of 3D-DIC measurements with projected speckle patterns, image acquisition by dual cameras captures light that has traversed through different optical paths, resulting in different thermal airflow distortions in the left and right images. Furthermore, the light projected by the projector is also affected by the thermal airflow; after being projected onto the surface of the sample and subsequently captured by the cameras, the path of the light, which traverses the thermal airflow turbulence zones twice, induces significant distortions in the final speckle images captured by the cameras. These distortions lead to deviations and potential errors in subset matching during DIC calculations, significantly compromising the accuracy of the 3D-DIC technique in evaluating the thermal reliability of microelectronic packaging structures.

To mitigate the influence of thermal airflow disturbances on 3D-DIC measurements, extensive efforts have been undertaken by the research community, encompassing both enhancements in hardware and advancements in algorithms, with the objective of increasing the accuracy and reliability of these measurements. A common hardware-based approach for eliminating the interference caused by turbulent thermal airflow involves employing devices such as fans and air knives to disperse the turbulent airflow, thereby stabilizing the airflow around the sample^21,22^. Berny et al.^23^ utilized a fan to remove part of the displacement fluctuations when studying the thermomechanical properties of ceramic matrix composites at temperatures above 1200 °C, increasing the accuracy of their measurements. Novak MD et al.^24^ employed an air knife to mitigate the effects of thermal airflow on measurements of composite materials at high temperatures, reducing the standard deviation (SD) of the measured strain by approximately threefold. Nonetheless, these approaches have proven insufficient for completely negating the effects of thermal airflow within the precision tolerances acceptable for microelectronic packaging reliability assessment. Furthermore, the integration of airflow management equipment, such as fans within the furnaces used for thermal warpage tests of microelectronic packaging structures, presents considerable challenges, not only in increasing experimental costs but also inadvertently affecting both heating and measurement processes.

Given these challenges, numerous algorithmic approaches have been developed to mitigate or counteract thermal airflow effects. A common method involves capturing several images of the sample, which are then averaged to create a single composite image^25,26^, or alternatively, by lengthening the exposure time to average the images directly^27^. Owing to the random nature of thermal airflow disturbances, this averaging method effectively reduces some of the turbulence, thus lessening its impact on the final measurement outcomes. However, this approach does not completely negate the effects of thermal disturbances unless a large series of images is captured over an extended period, which inevitably increases the duration of the measurement process and affects the accuracy of the results. A new correction technique based on deep learning, proposed by Liu et al.^28^, employs a network called the TSR-WGAN (temporal-spatial residual perceiving Wasserstein generative adversarial network; GAN) to correct images impacted by thermal airflows before DIC analysis is conducted, improving the measurement accuracy. Nevertheless, the relatively low heating temperatures in microelectronic packaging structures lead to minor distortions, posing challenges to the TSR-WGAN model, which is more effective in correcting significant distortions. Therefore, finding effective solutions specifically designed to overcome the challenges posed by thermal airflow disturbances in warpage measurements of microelectronic packaging structures remains a challenge.

To address the issue of low accuracy in thermal warpage measurements caused by thermal airflow disturbances during reliability testing of microelectronic packaging structures, inspired by the state-of-the-art ASF model in the field of atmospheric turbulence correction^29^, this paper introduces a neural network architecture based on the mechanism of the ASF model to correct thermal airflow distortions in images captured by cameras, followed by DIC calculations on the corrected images. The prevailing deep-learning methods for removing thermal airflow rely on GANs. While GANs are capable of producing images with rich details, they lack physical constraints, making it challenging to obtain reliable results^30^. When using convolutional neural networks (CNNs) to mitigate the effects of thermal airflow, the model tends to prioritize global consistency during the learning process, often at the expense of preserving high-frequency details in the image^31^. This approach may lead to the loss of high-frequency details such as speckle patterns, resulting in excessive smoothing of the image. Such oversmoothing is undesirable in scenarios where DIC calculations are based on speckle patterns. The image correction method proposed in this paper, which is based on the ASF framework, employs the alternating learning in spatial and frequency (LASF) mechanism to learn the temporal- and frequency-domain changes in the light field propagation process within thermal airflow, adaptively mitigating the adverse effects of thermal airflow. Furthermore, the incorporation of the squeeze-and-excitation (SE) attention mechanism enables the ASF model to better understand and utilize significant features of the input data, thereby enhancing the model’s performance and generalizability^32^; this also reduces the model’s complexity and further improves the quality of the reconstructed images, endowing the ASF model with the ability to restore complex textures, making it suitable for DIC measurements involving speckle patterns. This work presents a quantitative evaluation of the ASF model in an experimental setting for microelectronic reliability testing by measuring the 3D topography of a step block under thermal airflow disturbance. The experimental results demonstrate that the ASF model effectively enhances the quality of the captured images. When the DIC algorithm is used on the corrected images, the accuracy of the topography measurement meets the requirements of the microelectronic reliability testing domain, indicating potential practical application value in this field. Additionally, actual thermal warpage measurement experiments are conducted on packaged chips to further validate the practicality of the proposed method.

## Results

### The impact of thermal airflow disturbance on the measurement results

To demonstrate that the use of speckle-based 3D-DIC methods for measuring the thermal warpage of microelectronic packaging structures can result in accuracy loss due to thermal airflow disturbances, thereby underscoring the significance of this study, thermal warpage measurement experiments are conducted on alumina ceramic plates, as shown in Fig. 1.

**Fig. 1:**
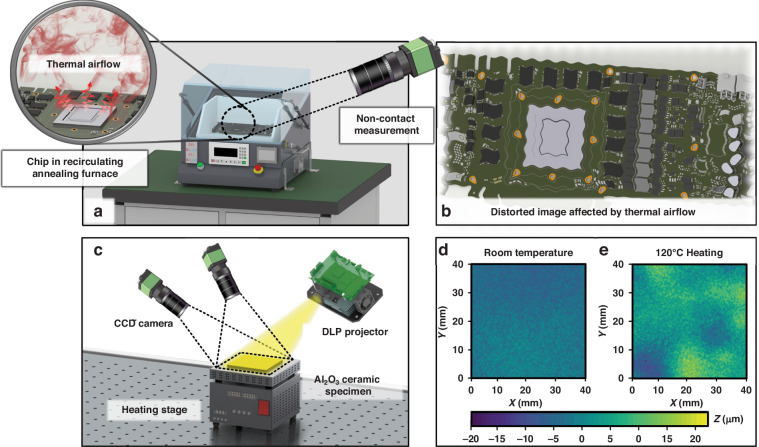
Schematic illustration of the impact of thermal airflow on DIC warpage measurements. **a** DIC measurement system capturing a chip within a heating furnace. **b** Distortions in the sample images captured by the DIC system, induced by thermal airflow effects. **c** Topographical measurement of an alumina ceramic substrate under thermal airflow disturbance. **d** Comparison of the measurement results with and without thermal airflow disturbance

As depicted in Fig. 1a, the packaging structure subjected to thermal experiments in a furnace experiences certain distortions in images captured via the DIC method for noncontact measurement of warpage. This distortion is attributed to the turbulent form of thermal airflow, as illustrated in Fig. 1b. To elucidate the impact of such image distortions on DIC measurement outcomes, a topographical measurement is conducted on an alumina ceramic plate under heating conditions, with the experimental setup shown in Fig. 1c. The measurement results without heating and at 120 °C are presented in Fig. 1d, e, respectively. Given the low coefficient of thermal expansion for the alumina ceramic (7.2 × 10^−6^/°C) and the use of a uniform alumina ceramic thin plate (42 mm × 42 mm × 3 mm) as the sample, the change in topography from room temperature to 120 °C is minimal. Therefore, it is presumed that the topography measured at 120 °C should be very close to that at room temperature. However, the measured topography results contain a significant number of abrupt height changes, which do not align with the actual situation. Thus, it is reasonable to attribute these discrepancies to errors introduced by thermal airflow disturbances causing image distortions. Owing to the different optical paths traversed by the light captured by the left and right cameras, the forms of image distortion vary, leading to a substantial number of subset mismatches; this introduces greater errors than those encountered with single-camera 2D-DIC, underscoring the impact of thermal airflow disturbances on measurement accuracy.

### Neural network structure for thermal airflow distortion image correction

To address the deficiency of other network models in preserving the complex textures of speckle patterns while eliminating thermal airflow distortions, this research enhances the ASF model on the basis of the transformer attention mechanism. This optimized model is more adept at correcting thermal airflow distortions in speckle images captured in the domain of microelectronic packaging structure warpage measurements because of its advantage in retaining the grayscale gradient changes of the speckle patterns in the images. The flowchart of the measurement system is depicted in Fig. 2.

**Fig. 2:**
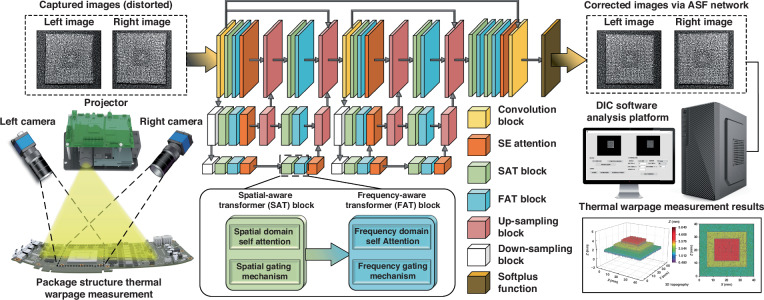
Flowchart of the DIC measurement method for correcting thermal airflow disturbances in microelectronic packaging structure warpage via a deep learning model

The model comprises two structurally identical multiscale models and one refinement module, each multiscale module consisting of three parallel branches. The first branch directly processes speckle images captured via stereo vision. The second branch receives input feature maps from the first branch, which are then downsampled by a factor of 2, and the number of channels is doubled. Downsampling helps capture the texture and shape features of adjacent pixels, reducing the volume of image data that must be processed; this enables the model to understand the global structure of speckle images while accelerating the processing speed. Increasing the number of channels enhances the model’s feature representation capacity. The third branch operates similarly to the second, but it downsamples the input features by a factor of 4, and the number of channels is quadrupled. Within each branch, feature maps are upsampled by a factor of 2 at the midpoint and at the end, with the number of channels halved; this helps to restore the image resolution while reducing the feature dimensions to control the model’s complexity. After these processes, the upsampled and compressed feature maps produced by each branch are fused with the feature maps from the previous branch. This fusion aids in integrating features captured at different scales, thereby enhancing the model’s understanding of the details and structure of speckle images. Finally, the images corrected by the neural network model are passed to DIC calculation software to compute the three-dimensional topography results of the sample.

### Principles and mechanisms

The LASF (learning alternating between spatial and frequency) mechanism is at the core of the ASF model depicted in Fig. 2. LASF, which is grounded in the self-attention mechanism, not only alternates the processing of speckle image data between the spatial and frequency domains but also deeply integrates the advantages of both domains. In the spatial domain, the spatial gating mechanism along with the GELU activation function are introduced, allowing the model to better handle and comprehend local details and spatial information in images. In the frequency domain, the frequency gating mechanism, together with the GELU activation function, is implemented, enabling the model to optimize the global consistency and structural information of images by learning which frequency information to retain. This complementary alternating learning strategy facilitates more accurate and robust image correction.

The input features are denoted by $X$. The frequency-aware transformer block is defined as Eq. (1):

1 $${Y}_{f}={P}^{-1}({{ {\mathcal F} }}^{-1}({ {\mathcal F} }(P({F}_{q}))\odot { {\mathcal F} }(P({F}_{k}))))$$

In the formula, ${ {\mathcal F} }$ denotes the Fourier transform, and ${{ {\mathcal F} }}^{-1}$ represents the inverse Fourier transform. $P$ refers to the operation of partitioning features, whereas ${P}^{-1}$ denotes the merging of partitioned features. ${F}_{q}$ and ${F}_{k}$ are the query and key features obtained from the linear transformation of the input feature $X$. The symbol $\odot$ signifies elementwise multiplication, and ${Y}_{f}$ is the attention map derived through operations in the frequency domain. Finally, the value feature is combined with the attention map ${Y}_{f}$, and through residual connection, the final frequency-domain self-attention feature map ${{Y}_{f}}^{att}$ is obtained, as shown in Eq. 2.

2 $${{Y}_{f}}^{att}=Conv(L({Y}_{f}){F}_{v})+X$$

Here, $Conv$ represents the convolution operation, $L$ denotes the normalization operation, and ${F}_{v}$ is the value feature obtained via linear transformation of the input feature $X$.

The spatial-aware transformer block is defined as follows:

3 $${{Y}_{s}}^{att}=Conv({S}_{v}\cdot Softmax({{S}_{k}}^{T}{S}_{q}/\alpha ))+X$$

Here, ${S}_{q}$, ${S}_{k}$, and ${S}_{v}$ represent the query, key, and value features obtained from the layer-normalized feature map $L(X)$ through depthwise separable convolution, respectively. ${{S}_{k}}^{T}$ denotes the transpose of ${S}_{k}$, while the $Softmax$ function normalizes the input scores, converting them into a probability distribution suitable for information perception. The parameter $\alpha$ is used for scaling.

High-frequency details are closely associated with the sharpness and textural information of an image. To enhance the texture information in images, this study introduces a loss function named the patch Fourier transform loss ${L}_{patchfft}$. This function encourages the ASF model to focus more on high-frequency details by comparing the differences between the frequency-domain representations of the transformed and original images.

Additionally, this paper incorporates the SE self-attention mechanism into the ASF model, which dynamically recalibrates feature channels. This mechanism serves to enhance the model’s focus on salient features, thereby improving the quality of image restoration. To circumvent the risk of overfitting and curb computational demands, and considering that features in the frequency domain generally encompass the overall structure of the image, the SE module is applied exclusively after the FAT module in specific positions.

### Experimental setup

To verify the effectiveness of the proposed neural network-based DIC measurement method (N-DIC) in correcting thermal airflow disturbances and enhancing the accuracy of DIC measurements for microelectronic packaging structure topography, a customized step block topography measurement experiment was conducted, as illustrated in Fig. 3. In the DIC topography measurement process, larger errors often occur at height transitions than in flat areas; thus, using step block topography allows for a better assessment of the measurement accuracy of N-DIC.

**Fig. 3:**
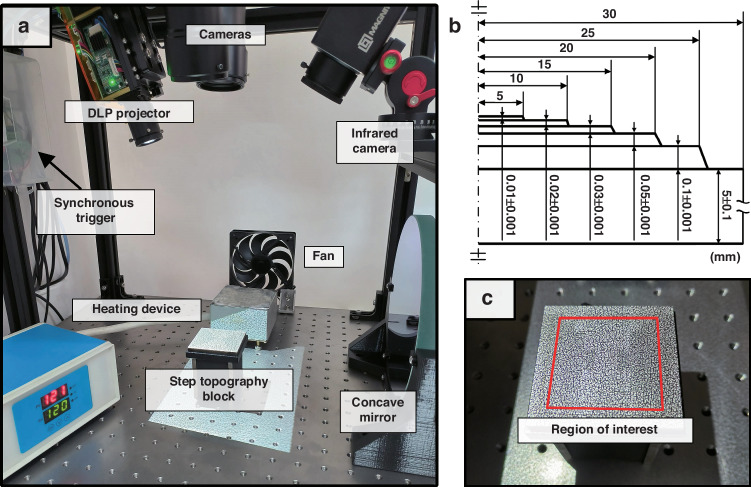
Physical setup of the step block topography measurement experiment. **a** Experimental setup layout. **b** Dimensional diagram of the customized step topography block used. **c** Physical image of the topography block covered with speckles and a schematic diagram of the selected region of interest (ROI)

As shown in Fig. 3a, the experimental setup is placed on an optical vibration isolation platform, with a DLP projector used to project software-generated speckle patterns onto the sample to be tested and a dual-camera system arranged to capture images of the sample. Figure 3b, c display the dimensions of the step block sample and an actual image of the sample covered with speckles, respectively. The ROI includes the top four steps of the block, which have smaller height differences and are thus better indicators of the accuracy of the topography measurements.

A temperature-controlled heating device is used to generate thermal airflow, with a variable-speed fan directing the airflow over the topography block to simulate the thermal air currents produced by microelectronic packaging structures during heating. To replicate the actual conditions of packaging structures within a heating furnace, where the thermal airflow creates turbulent disturbances above the topography block, a concave mirror is positioned behind the block for Schlieren image capture. This setup aids in determining the appropriate fan speed by visualizing the airflow patterns.

To ensure that the topography block itself is not influenced by the heating device or thermal airflow, leading to thermal expansion that could alter its original form and compromise the reliability of the experimental results, an infrared camera is employed to monitor the temperature of the topography block. This measure ensures that the temperature remains constant, allowing us to assume that the three-dimensional topography of the block remains unchanged throughout the experiment.

### Determination of fan speed

To ensure that the thermal airflow at different temperatures can cover the area above the topography block, causing deformation interference to the collected images and thus simulating real conditions, it is necessary to determine the appropriate fan speed. To observe whether thermal airflow formed above the topography block, schlieren experiments are conducted using a concave mirror, light source, blade, and camera. The experimental setup and optical path are illustrated in Fig. 4a, b.

**Fig. 4:**
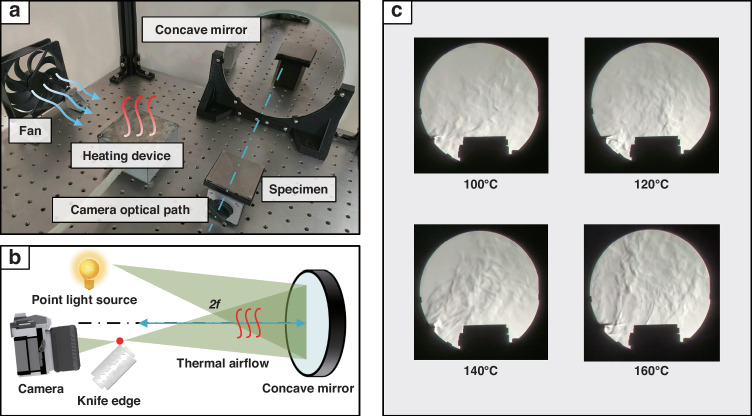
Experiment for determining fan speed. **a** Physical setup of the experiment. **b** Diagram of the experimental optical path. **c** Captured thermal airflow above the sample at different temperatures

Using a heating device, thermal airflow at temperatures of 100 °C, 120 °C, 140 °C, and 160 °C are generated. Once the temperature stabilizes, the fan is turned on, and its speed is controlled via pulse width modulation to ensure that the thermal airflow can form above the topography block, causing deformation interference in the images captured by the camera and thus simulating the actual conditions of microelectronic packaging structures during heating. When the fan speed reaches 750 rpm, the thermal airflow can be directed above the sample, as shown in Fig. 4c.

### Topography measurement experiment of the topography block under thermal airflow disturbance

The topography of the sample block is measured via a projected speckle-based 3D-DIC measurement system. Images captured synchronously by the left and right cameras were stored on a computer, corrected via the neural network, and then used to produce corrected images for both cameras. DIC calculations are subsequently performed to obtain the topography results of the sample. A total of five experimental groups are conducted, corresponding to no thermal airflow disturbance and thermal airflow disturbances at 100 °C, 120 °C, 140 °C, and 160 °C. The fan speed is determined on the basis of previously described fan speed experiments. For comparison, images distorted by thermal airflow disturbances that were not corrected by the neural network are also directly subjected to DIC calculations, allowing for the quantification of the precision improvement afforded by the proposed method. Additionally, the distorted images are corrected via NAFNet, which was proposed by Chen et al.^33^, and the EAF-WGAN proposed by Liu et al.^34^, followed by DIC calculations to assess the errors. This evaluation is conducted to determine the accuracy of the N-DIC method.

To obtain accurate three-dimensional topography of the customized step block, a laser scanning profilometer is used to scan it, with the acquired topography results serving as the ground truth. Because the step block sample in the experiment is not heated directly but instead has thermal airflow formed above it via a fan, the temperature of the sample remains constant, and no thermal deformation occurs. Therefore, the 3D topography of the sample obtained from laser scanning can be used as the true topography under different temperatures to calculate the errors of the DIC and N-DIC methods. The measurement profiles of the DIC and N-DIC results at the same profile positions are subtracted from the corresponding profile height values obtained via the laser scanning method to generate error curves. These error curves reflect the deviations of the DIC and N-DIC methods relative to the laser scanning results, thereby providing an assessment of the measurement accuracy of the DIC and N-DIC methods.

### Thermal warpage testing for microelectronic packaging specimens

To demonstrate the effectiveness of the proposed neural N-DIC method in reducing thermal airflow disturbances and improving precision through step block specimen experiments, thermal warpage testing is carried out on microelectronic packaging chip samples. Different temperature loads are applied to the microelectronic packaging samples, as depicted in Fig. 5. The N-DIC method is utilized to detect their thermal warpage conditions, further extending the method’s applicability from theoretical validation to practical testing in the field of microelectronic packaging reliability.

**Fig. 5:**
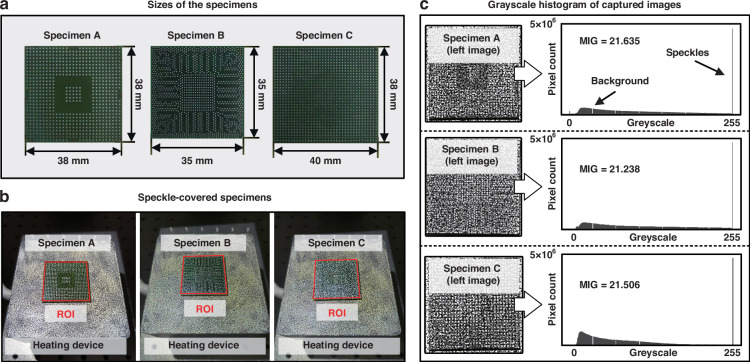
Microelectronic packaging chip samples for thermal warpage testing. **a** Dimensions of the microelectronic packaging chip sample. **b** Actual image of the sample covered with speckle patterns during thermal warpage measurement. **c** Grayscale histogram of the sample image captured by the CCD camera (using the image from the left camera as an example)

Figure 5a displays the dimensions and actual image of the measured sample, from which the solder balls are removed prior to the experiment. Figure 5b shows the sample, which is placed on a heating stage and projected with speckle patterns, highlighting the ROI for subsequent calculations. Figure 5c presents an image captured by the CCD camera along with a grayscale histogram of the captured image (using the left camera as an example, with the right camera having similar parameters and nearly identical grayscale statistics). The histogram shows high grayscale values in the areas of the speckle patterns and solder pad regions, whereas the lower grayscale values correspond to the substrate area (background). The grayscale histogram demonstrates a good gradient of grayscale changes, ensuring that subsequent DIC calculations are not compromised by insufficient speckle contrast, which could lead to subset matching failures. Additionally, the mean intensity gradient^35^ of the image is calculated to further verify that the speckles in the captured image have sufficient contrast.

### Measurement results

To demonstrate the versatility of the proposed N-DIC method in measuring specimen warpage, it is essential to test whether the correction model introduces new errors due to overcorrection when there are no thermal airflow disturbances in the input images. Therefore, the topography of the step block specimen is measured via both the standard DIC method and the N-DIC method in the absence of thermal airflow disturbances. The results are then compared with the laser-scanned topography to assess the measurement errors, with the outcomes depicted in Fig. 6.

**Fig. 6:**
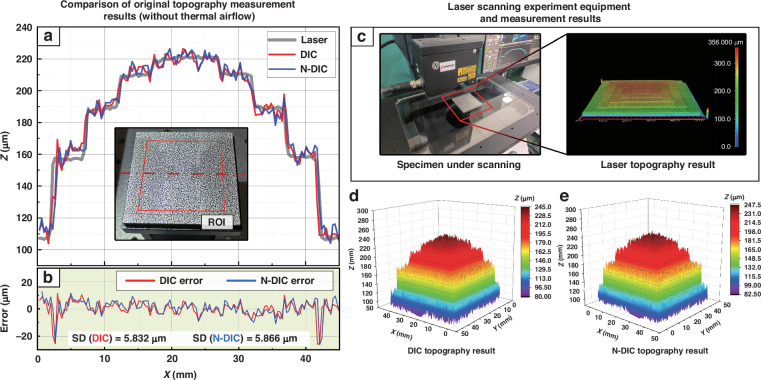
Comparison of standard DIC and N-DIC measurement errors without thermal airflow disturbance. **a** Comparison of the topography at the central cross section obtained via DIC, N-DIC, and laser scanning methods. **b** Errors in the DIC and N-DIC measurements relative to the laser scanning results. **c** Laser scanning experimental equipment and three-dimensional measurement results. **d** Three-dimensional topography results measured via DIC. **e** Three-dimensional topography results measured via N-DIC

As shown in Fig. 6, the topography results of the specimens measured via both the standard DIC and N-DIC methods exhibit high consistency when unaffected by thermal airflow. A comparison of the profiles in Fig. 6a indicates that both the conventional DIC method and the N-DIC method precisely capture and reflect the step-like structure of the specimen and the height of each step (using the central cross-sectional topography as a representative example).

By taking the laser scanning results as the true values, the discrepancy between the DIC and N-DIC results and the laser scanning data was calculated, as shown in Fig. 6b. The results show that, except for a few significantly deviated outliers, the measurement error is within ±10 μm. Specifically, the SD for the DIC method is 5.832 µm, and the SD for the N-DIC method is 5.866 µm, with no significant difference between the two SDs. This proves that in the absence of thermal airflow interference, the correction neural network in the N-DIC method does not introduce new errors by misjudging and incorrectly correcting undistorted images; this demonstrates the accuracy of the N-DIC method in determining the presence of thermal airflow distortion and proves the robustness of the N-DIC method when measuring specimen warpage. Therefore, in the measurement of the thermal warpage of microelectronic packaging structures, the N-DIC method does not require a prior determination of the presence of thermal airflow disturbances in the images and can be directly applied in actual measurements, which is highly valuable for simplifying experimental procedures and practical applications.

When subjected to thermal airflow disturbances at various temperatures, the topography of the step block specimen is measured, and the discrepancies relative to the laser scanning data are evaluated (using the central cross-sectional topography as a representative example). The results are presented in Fig. 7.

**Fig. 7:**
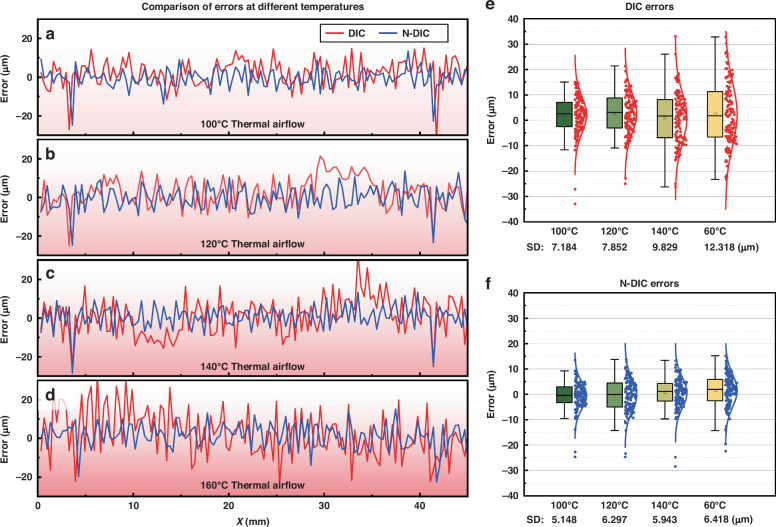
Measurement errors of the DIC and N-DIC methods under thermal airflow disturbance at different temperatures. **a**–**d** Comparison of measurement errors between the DIC and N-DIC methods under thermal airflow disturbances at 100 °C, 120 °C, 140 °C, and 160 °C. **e** Statistical overview of measurement errors with the DIC method. **f** Statistical overview of measurement errors with the N-DIC method

Figure 7 clearly demonstrates that thermal airflow significantly increases measurement errors in the DIC method, especially at higher temperatures. The statistical data in Fig. 7e indicate that the errors introduced by thermal airflow increase with increasing temperature. This observation can be attributed to the intensified strength and turbulence of the thermal airflow at elevated temperatures, resulting in greater image distortions, thereby causing more severe errors in DIC calculations and even incorrect subset matching. However, the application of the N-DIC method substantially reduces these errors, as shown in Fig. 7f, underscoring the effectiveness of the proposed N-DIC method in correcting for thermal airflow disturbances. Figure 7a–d presents the error comparison between the DIC and N-DIC methods at various temperatures, with the N-DIC method maintaining lower error levels across all temperatures. Additionally, the DIC measurement results reveal segments with significantly larger error values, particularly at temperatures of 120 °C, 140 °C, and 160 °C. This phenomenon is attributed to the instability and uneven distribution of thermal airflow turbulence, which forms larger heat-haze clusters in certain regions, leading to greater distortions and causing substantial deviations in the DIC measurement results. However, in the corresponding regions of the N-DIC measurement results, the errors are not significantly elevated. This finding demonstrates that the N-DIC method can effectively correct distortions and reduce measurement errors, even in the presence of pronounced thermal airflow distortions. In summary, the N-DIC method effectively diminishes the impact of thermal airflow disturbances on measurements, thereby enhancing measurement accuracy, especially at high temperatures. This finding has significant practical implications for the measurement of thermal warpage in microelectronic packaging.

Additionally, the error statistics for image distortion correction and DIC measurements via the CNN-based model (NAFNet)^33^ and the GAN-based model (EAF-WGAN)^34^ are shown in Table 1.

**Table 1:** Standard deviation of the measurement errors for different heat-haze correction models

Methods	SD/μm			
	100 °C	120 °C	140 °C	160 °C
DIC	7.184	7.852	9.829	12.318
N-DIC	5.148	6.297	5.943	6.418
CNN-Based	6.653	7.377	8.956	10.755
GAN-Based	6.371	7.135	7.812	8.986

As shown in Table 1, the measurement errors are more significant when the CNN-based and GAN-based models are used than when the N-DIC model based on the ASF framework is used; this is because the CNN-based model smooths out the image details during heat-haze distortion correction, which is detrimental to speckle matching and correlation calculations. Similarly, the GAN-based model generates images with a certain number of speckle shapes that do not match the original image, leading to greater measurement errors than those of the N-DIC model.

Thermal warpage measurement experiments are conducted on three microelectronic packaging specimens, A, B, and C, with the results shown in Fig. 8 (represented by the topography changes along the diagonal). The DIC measurement results in Fig. 8a–c indicate that as the heating temperature increases, the fluctuations in the thermal warpage measurement results become more intense, indicating the impact of thermal airflow distortion on the measurements. Additionally, because the DIC measurement system used in this study employs projected speckles, the light from the projector is affected by the thermal airflow before it is projected onto the sample. The light is then further disturbed by the thermal airflow along different optical paths before being captured by the cameras; this results in significant errors and considerable fluctuations in the measurement results. After the N-DIC method is used for distortion correction, as shown in Fig. 8d–f, the warpage measurement results are smooth and continuous, which is consistent with the continuous and uniform reality of sample thermal warpage; this demonstrates the practicality of the N-DIC method in detecting actual thermal warpage in microelectronic packaging samples via projected speckles.

**Fig. 8:**
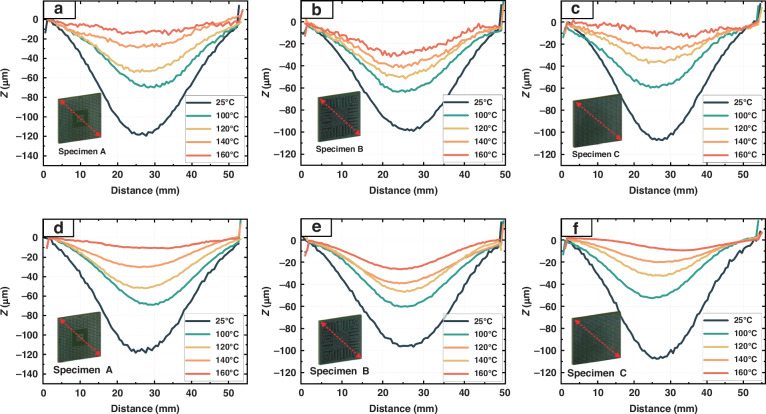
Thermal warpage measurement results for the microelectronic packaging samples. **a**–**c** Warpage measurement results for specimens A, B, and C via the DIC method. **d**–**f** Warpage measurement results for specimens A, B, and C via the N-DIC method

## Discussion

In the field of thermal warpage measurement for microelectronic packaging structures, the thermal airflow generated during the heating process introduces distortions in image acquisition, which disrupts the accuracy of noncontact measurements and reduces the reliability of measurements. To address this challenge, inspired by the ASF model used for atmospheric turbulence correction, this study proposes an optimized model tailored for the thermal warpage measurement domain of microelectronic structures. By correcting thermal airflow distortions in the acquired images, the precision of DIC measurements is enhanced. The proposed model integrates the transformer attention mechanism to avoid the oversmoothing issues commonly associated with conventional CNNs and the limitations of GANs, ensuring the accuracy of grayscale gradient changes in speckle patterns and preventing negative impacts on DIC computation accuracy caused by alterations in speckle details.

The model structure in this study is specifically designed and optimized for the application scenario of measuring thermal warpage in microelectronic packaging, ensuring that its functionality aligns closely with the required context. By incorporating the SE attention mechanism to merge multiscale information, the model’s ability to process key local features and global information in images collected during thermal warpage measurement of microelectronic packaging structures is enhanced, allowing for better understanding and reconstruction of the detailed structure of speckle images and making it more suitable for removing thermal airflow disturbance distortions in speckle images. Through topography measurement experiments on customized step blocks subjected to thermal airflow disturbances at different temperatures, this paper demonstrates the effectiveness and practicality of the improved model. Compared with traditional methods, the proposed improved model can significantly reduce measurement errors caused by thermal airflow, effectively enhancing measurement precision and reliability, especially at common temperatures for chip packaging structure thermal warpage tests such as 140 °C and 160 °C, where the standard deviations are reduced from 9.829 to 5.943 µm and from 12.318 to 6.418 µm, respectively.

Therefore, this study not only presents an innovative approach to improve the accuracy of thermal warpage measurements in microelectronic packaging structures but also provides a deep learning solution framework for specific measurement challenges. This framework has potential applications in the reliability measurement of actual microelectronic packaging products.

## Methods

### Experimental equipment

The experimental setup for the 3D-DIC measurement system utilizes a DLP projector, model DLP4500SL02, with a resolution of 1280 × 800 pixels. The CCD cameras for image acquisition are model DAHENG MER-500-7UM, featuring a resolution of 2592 × 1944 pixels and a frame rate of 7 fps, complemented by lenses with focal lengths ranging from 8.5 mm to 90.00 mm and a working distance from 300 mm to infinity. This setup forms a stereo measurement system to capture images of the specimen. The dual cameras use a synchronous trigger for hard triggering, ensuring synchronization accuracy within 5 ms. The infrared thermal imager used to monitor the sample’s temperature during the experiments is model MAG32, with a resolution of 384 × 288 pixels, a frame rate of 50 fps, and a temperature resolution within 60 mk. Both the image acquisition system and the speckle projection system are mounted on a gantry structure built from aluminum profiles on an optical vibration isolation platform, ensuring that the system is not affected by vibrations and thereby avoiding additional errors.

For the control group in the topography measurement experiments, a laser scanning profilometer, model CHOTEST-VJ2010, with a scanning repeatability accuracy of ±1 μm, is utilized alongside the associated measurement software VisionX Pro.

### Speckle pattern and subset configuration

The speckle pattern is computer-generated, featuring a black background with white speckles. The resolution of the speckle image is 2000 × 2000 pixels, with an average speckle diameter of 3.5 pixels and a standard deviation of 0.25 pixels for the diameter. The speckle image is projected onto the sample and then captured via CCD cameras. By adjusting the scaling percentage of the projected image, 3 to 5 speckles are formed within each subset in the image captured by the CCD camera. The subset size is set to 31 × 31 pixels, with a step size of 15 pixels to ensure that DIC calculations can be performed across all ROIs.

### Supplementary information

SUPPLEMENTAL MATERIAL
