# Supplementary material for: Correction of thermal airflow distortion in warpage measurements of microelectronic packaging structures via deep learning-based digital image correlation
Source: Microsyst Nanoeng. 2024 Aug 26;10:118. doi: 10.1038/s41378-024-00764-8 (PMC11347651; doi:10.1038/s41378-024-00764-8)
Supplement: Supplementary file 1 — SUPPLEMENTAL MATERIAL [file 41378_2024_764_MOESM1_ESM.docx]

Supplementary information

# Correction of Thermal Airflow Distortion in Warpage Measurements of Microelectronic Packaging Structures Using Deep Learning-Based Digital Image Correlation

Yuhan Gao^1^, Yuxin Chen^1^, Ziniu Yu^1^, Chuanguo Xiong^1^, Xin Lei^1^, Weishan Lv^1^,

Sheng Liu^1,2^, Fulong Zhu^1^

^1^Institute of Microsystems, School of Mechanical Science and Engineering, Huazhong University of Science and Technology, Wuhan, 430074, China

^2^School of Power and Mechanical Engineering, Wuhan University, Wuhan 430072, China

Corresponding Author: [zhufulong@hust.edu.cn](mailto:zhufulong@hust.edu.cn)

The experimental setup incorporated a DLP4500SL02 projector, which has a resolution of 1280×800 pixels. The CCD cameras used were of the DAHENG MER-500-7UM model, featuring a resolution of 2592×1944 pixels with a capture rate of 7 frames per second. The optical system utilized lenses from Edmund Optics, specifically model #68-679, with a focal length adjustable from 8.5mm to 90mm and a working distance ranging from 300mm to infinity. In the experiments, horizontal Field of View (FOV) is approximately 16.80°.

Table S1. Reprojection error of two cameras.

| Sets | Left camera(C1) and right camera(C2) |
| --- | --- |
| 1 | 0.0967 |
| 2 | 0.1115 |
| 3 | 0.1274 |
| 4 | 0.1371 |
| 5 | 0.1275 |
| 6 | 0.1312 |
| 7 | 0.1242 |
| 8 | 0.1361 |
| 9 | 0.1256 |
| 10 | 0.0960 |
| 11 | 0.1599 |
| 12 | 0.1587 |
| 13 | 0.1488 |
| 14 | 0.1365 |
| 15 | 0.1347 |
| 16 | 0.1523 |
| 17 | 0.1188 |
| 18 | 0.1492 |
| 19 | 0.1255 |
| 20 | 0.1142 |
| 21 | 0.1194 |
| Average | 0.1301 |

Reprojection error of twenty-one sets of calibration board images.

The comparison of speckle images of the specimen before and after correcting the heat haze distortion is shown in Figure. S1 (example with the left camera image under 140℃).


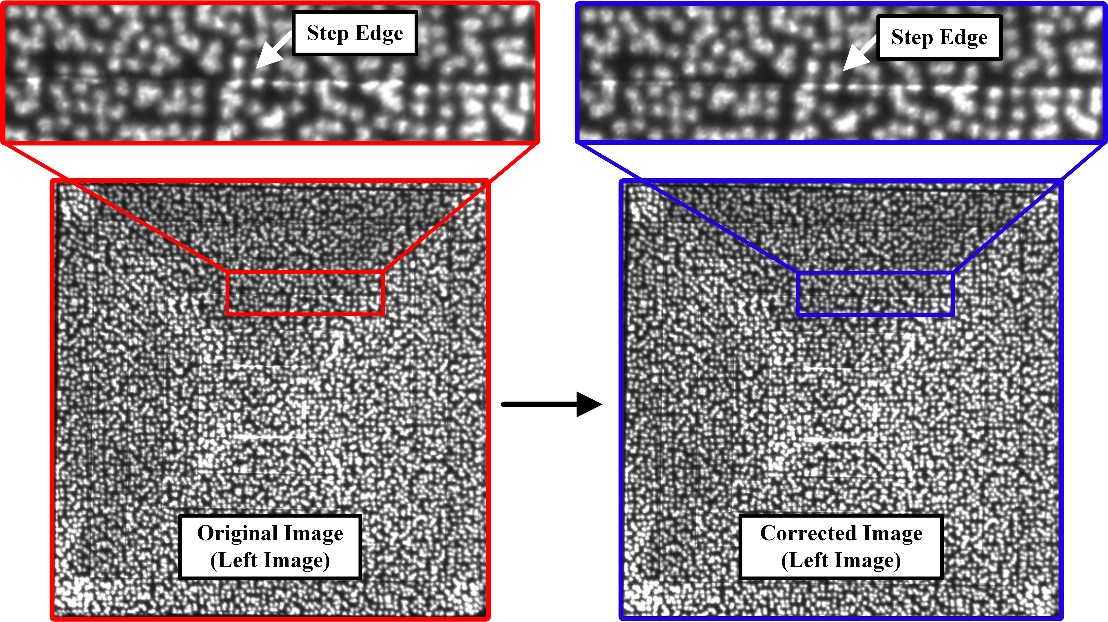


Figure. S1: Comparison of speckle-covered specimen images before and after correction (example with the left camera image under 140℃ heat haze disturbance)

From Figure. S1, it is evident that the heat haze disturbance causes the edges of the step block specimen in the original images to appear irregular. In contrast, the edges in the corrected images are noticeably straighter and align more closely with the actual specimen. This provides a clear visual demonstration of the N-DIC correction capabilities.
